# An internet‐delivered self‐management programme for bipolar disorder in mental health services in Ireland: Results and learnings from a feasibility trial

**DOI:** 10.1002/cpp.2480

**Published:** 2020-06-10

**Authors:** Angel Enrique, Daniel Duffy, Kate Lawler, Derek Richards, Steven Jones

**Affiliations:** ^1^ E‐mental Health Research Group, School of Psychology University of Dublin, Trinity College Dublin Ireland; ^2^ Clinical Research & Innovation SilverCloud Health Dublin Ireland; ^3^ Spectrum Centre for Mental Health Research University of Lancaster Lancaster UK

**Keywords:** acceptability, bipolar disorder, feasibility, implementation, internet‐delivered treatment, self‐management

## Abstract

Bipolar disorder (BD) is a chronic condition that requires continued care. Psychological interventions are recommended by clinical guidelines but there are treatment barriers that prevent patients to access these services. Internet‐delivered self‐management interventions are promising alternatives to improve treatment accessibility in patients with BD. Several studies indicate that these interventions are acceptable and beneficial for patients with BD, but no studies have been conducted in routine care settings. This trial aimed to examine the feasibility, acceptability, and preliminary efficacy of implementing an internet‐delivered, clinician‐supported intervention for BD as an adjunct to treatment as usual at two secondary‐care services in Ireland. This study used an uncontrolled design with mixed‐methods evaluation. Feasibility and acceptability were assessed in terms of recruitment, use of the intervention, and satisfaction from both clinicians and patients' perspectives. Personal recovery, quality of life, and severity of symptoms were measured at baseline and post‐intervention. Fifteen patients signed consent and used the programme for 10 weeks. Usage of the intervention was adequate with high frequency of tool usage. There was a significant improvement in patients' sense of personal recovery (*z* = 2.38, *p* = .017). The intervention was found acceptable and easy‐to‐use; however, implementation barriers will need to be overcome for scaling the intervention. This is the first study testing the feasibility of a digital intervention for patients with BD in public mental health services in Ireland. More research is needed in order to increase the understanding of how to promote the integration and the uptake of digital interventions for individuals with BD.

**Key Practitioner Messages**

- The internet‐delivered, self‐management intervention for bipolar disorder was offered along with the treatment as usual at two secondary‐care services in Ireland and was supported by on‐site clinicians for 10 weeks.
- The internet‐delivered intervention was developed in an iterative process with different stakeholders so that it covered the needs for patients and clinicians, and it was based in the personal recovery approach.
- Patients who used the intervention reported high levels of satisfaction and statistically significant improvements were observed in perceptions of personal recovery, but no changes were noted on quality of life and symptom severity.
- Clinicians who offered the support found the intervention easy‐to‐use and the content helpful, but they were not able to provide the intended amount of reviews due to significant workload.
- Implementation factors need to be considered in order to achieve a proper deployment of digital interventions into mental health services.

## 1 INTRODUCTION

Bipolar disorder (BD) constitutes a potentially chronic and disabling condition characterized by extreme fluctuations in mood including recurrent episodes of either depression, mania, or mixed affective states (Grande, Berk, Birmaher, & Vieta, 2016). BD affects approximately 2% of the population worldwide (Merikangas et al., 2011), and a similar rate has been observed in England (McManus, Bebbington, Jenkins, & Brugha, 2016), with societal costs of £4.59 billion only in the United Kingdom (Fajutrao, Locklear, Priaulx, & Heyes, 2009). BD generally starts in young adulthood with the average duration from illness onset to its correct diagnosis ranging from 5 to 10 years (Berk et al., 2007).

The National Institute for Health and Care Excellence (2014) recommends a combination of both pharmacological and psychological interventions for an adequate management of BD. Consistent with this, two recent meta‐analyses demonstrate that psychological interventions can reduce relapse rates and the severity of depressive and manic episodes, along with increases in psychosocial functioning (Chiang et al., 2017; Oud et al., 2016). However, the effect sizes are not large and the underlying treatment mechanisms are not fully understood suggesting that more research is needed to improve outcomes for people with BD (Murray et al., 2017). Furthermore, patients with severe mental conditions, such as bipolar, have expressed dissatisfaction with clinical recovery being the primary outcome of clinical practice (Jones et al., 2010; Mead & Copeland, 2000). Thus, in the last decade, government policies are recognizing the importance of personal recovery outcomes, which are more focused on improving the quality of life and having personally meaningful goals (Cavelti, Kvrgic, Beck, Kossowsky, & Vauth, 2012; Murray et al., 2017).

Despite the benefits of psychosocial interventions, there is an enormous gap between demand and availability of treatment that prevent patients to access treatments (Harvey & Gumport, 2015; Mohr et al., 2010). This situation is specially difficult in individuals with BD, because most of the services deliver face‐to‐face episodic care and are not configured to meet the chronic and fluctuating needs of these patients (National Institute for Health and Care Excellence, 2014; World Health Organization, 2013). Therefore, there is a need for innovative solutions that help to overcome treatment barriers. Internet‐delivered interventions offer the potential to facilitate access to resources that promote recovery and symptom management, while reducing waiting lists and delivery costs (Wallin, Mattsson, & Olsson, 2016). These interventions have already shown effective and practical deliver mental health treatments for depression and anxiety disorders (Andrews et al., 2018; Wright et al., 2019).

During the last decade, various internet‐delivered interventions for BD have been developed, and most of them have been proposed as an adjunct to treatment as usual. These initiatives have been based on psychoeducation about BD (Barnes & Simpson, 2011), along with cognitive‐behavioural therapy (Lauder et al., 2015; Proudfoot et al., 2012; Todd, Jones, Hart, & Lobban, 2014), mindfulness (Fletcher et al., 2018; Murray et al., 2015), and interpersonal and social rhythm therapy (Lieberman, Swayze, & Goodwin, 2011). Regardless of the psychological approach, ongoing monitoring, education, and planning and taking action were the most common strategies included in digital interventions for bipolar (Gliddon, Barnes, Murray, & Michalak, 2017). Furthermore, some of the interventions also included support from patients' peers or coaches that resulted in higher rates of acceptance and retention (Gliddon et al., 2019; Proudfoot et al., 2012; Todd et al., 2014). Lastly, a recent initiative tested a self‐management intervention aimed at fostering parenting skills for parents with BD (Jones et al., 2017).

In general, the different initiatives have shown high rates of acceptability, satisfaction, and retention, suggesting that they are a feasible method for delivering interventions (Gliddon et al., 2017; Hidalgo‐mazzei et al., 2015). In terms of the efficacy of these interventions, the observed effects to date are mixed, where some studies have observed improvements in quality of life (Murray et al., 2015) and mood severity (Lauder et al., 2015; Lieberman et al., 2011), whereas others have found no significant effects (Barnes, Hadzi‐Pavlovic, Wilhelm, & Mitchell, 2015; Proudfoot et al., 2012; Todd et al., 2014). Most of these studies were conducted in community settings or with participants from other clinical settings (Barnes et al., 2015; Hidalgo‐mazzei et al., 2016; Lobban et al., 2017), where the recruitment of participants and the intervention procedures were administered independently of the clinical service and using external staff resources, which limits the learnings about how these solutions may benefit current services provision (Hidalgo‐mazzei et al., 2015; Rabin & Browson, 2017).

Despite the mixed findings in terms of efficacy, many different experts agree on the positive impact and utility these interventions will have at the different levels of care for individuals with BD (Barnes et al., 2015; Murray, 2019). In the same vein, integrating these solutions into existing services and being supported by healthcare staff could be of substantial benefit (Aref‐adib et al., 2018; Hidalgo‐mazzei et al., 2015). At an organizational level, interoperability with existing systems and adaption of the solution to the local environment has been found as key factors for a successful implementation (Aref‐adib et al., 2018). Similarly, understanding the role that healthcare staff may play in the support of these interventions is of relevance, because it has been found that staff engagement from onset of the process is key for successful adoption (Koivunen, Hätönen, & Välimäki, 2008). To date, no studies have explored the feasibility of integrating digital interventions into exisiting service pathways for outpatients with bipolar, which partly may be due to the absence of suitable infrastructures to support the delivery of these interventions (Aref‐adib et al., 2018). This caveat in the literature prevents to understand how these pathways, the service users and staff could benefit from the use of these interventions. Thus, it is important to investigate the utility and barriers of integrating digital interventions into existing mental health services, especially secondary care services where these individuals are mostly treated.

The present study seeks to develop an internet‐delivered self‐management intervention for bipolar and explore the acceptability, feasibility, and preliminary clinical benefits of its integration as part of the care plan for patients with a diagnosis of BD at two secondary care services in Ireland.

The specific objectives were as follows:

1. To explore number of patients recruited during the research trial period.
2. To analyse the uptake of the intervention by patients in terms of usage of the intervention.
3. To explore acceptability and satisfaction of service users with the self‐management tool.
4. To quantitatively explore the outcomes of the internet‐delivered treatment on quality of life, recovery, and other related clinical symptoms.
5. To explore the uptake and engagement of the clinicians in providing the support.

## 2 METHODS

### 2.1 Trial design

The study utilized an uncontrolled, within‐group, pre‐post design with embedded mixed‐methods evaluation. All participants included in the trial were offered the internet‐delivered self‐management intervention for 10 weeks to be used as an adjunct to their treatment at usual (TAU).

### 2.2 Study setting

This project was launched as part of the eHealth Ireland strategy (Lighthouse project, https://www.ehealthireland.ie/Lighthouse-Projects/) developed by the Health Service Executive (HSE) that is responsible for the provision of health and social care services for the population of the Republic of Ireland. The solution was piloted and tested at two speclized secondary care services (Sites A & B) that provide services to individuals with severe mental disorders. Site B is considered a commuter town of the capital city, Dublin, whereas Site A was more rural in nature.

Ethical approval to conduct this study was granted by the Research Ethics Committee, HSE South‐Eastern Area (16/07/2018) and the Research Ethics Committee, Wicklow Mental Health (17/04/2018) to conduct research at the sites.

### 2.3 Sample size

As this naturalistic study was conducted in a public health setting, the support was provided by the clinicians working at each site. Therefore, the sample size depended on the weekly capacity clinicians at the site had to provide the support for the intervention. To reduce the burden on both of the sites in the study, we sought to recruit 10 patients per site to participate, resulting in a total of 20 participants. We expected each clinician to support a maximum of three patients, depending on the number of patients enrolled in the trial.

### 2.4 Eligibility criteria

#### 2.4.1 Patient elegilibity criteria

- Aged 18 years or over with access to a smartphone or computer and the internet.
- The capacity to provide consent to participate.
- Clinical diagnosis of BD Type I or II established by a psychiatrist.
- Not having an active depressive or manic episode determined by the psychiatrist on site
- No risk of suicide determined by the psychiatrist on site
- Not using other self‐management tools for bipolar, such as WRAP or similar tools.
- Not taking part in another intervention study (or follow‐up period).

#### 2.4.2 Clinician elegibility criteria

- Belonging to the clinical team in charge of patients with BD.
- Included any of the specialists in the care team (i.e., psychiatrist, doctor, nurses, social worker, psychologist, and occupational therapist).

### 2.5 The intervention—Bipolar Toolkit

#### 2.5.1 Intervention development

The content of the *Bipolar Toolkit* intervention was informed by a four‐stage development phase that involved input from a variety of sources. The development phases closely followed the guidelines of the UK Medical Research Council for the development of complex interventions (Craig et al., 2008). First, a review of the literature was conducted to explore existing evidence‐based self‐management tools for bipolar diagnosis, face‐to‐face, and internet‐delivered. This stage helped to identify the main components that a self‐management tool should include (Hidalgo‐mazzei et al., 2015; Leitan, Michalak, Berk, Berk, & Murray, 2015). Based on the literature review, the theoretical model that the intervention would be based on was identified, namely, the personal recovery approach (Higgins, 2008; Onken, Craig, Ridgway, Ralph, & Cook, 2007). This approach was indicated as the most suitable model for self‐management interventions developed for severe mental illness. Thereafter, several interviews were conducted with various stakeholders across the services involved in the project, including two service users, two clinical nurse managers, two psychiatrists, and one occupational therapist. The aim of the interviews conducted with the clinicians was to explore the needs that the intervention could meet from both, the clinician and patient perspective. Finally, the information gathered throughout previous phases was synthesized to develop a content map. This content map was used to create an alpha version of the programme, which was subsequently presented to stakeholders for a final review, and modified accordingly based on the feedback.

#### 2.5.2 Intervention description

**FIGURE 1:**
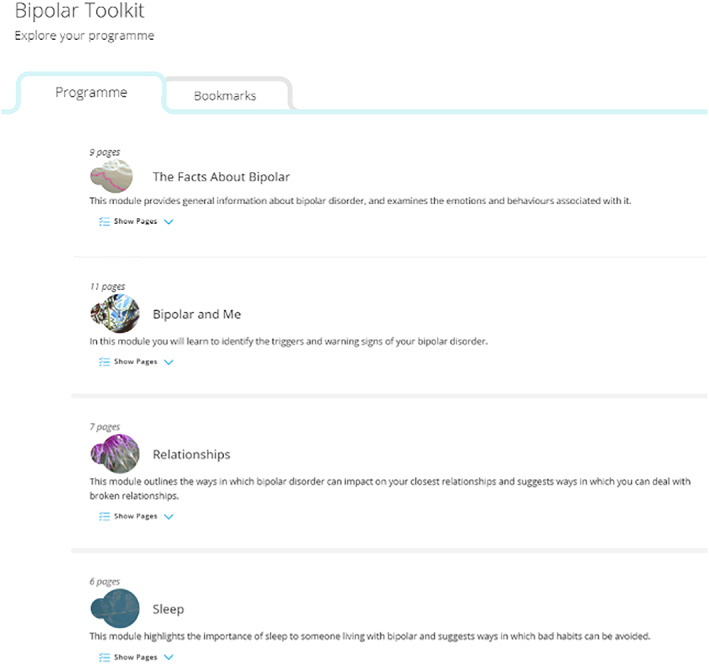
Screenshot of the user dashboard, illustrating the modules and a short description of each [Colour figure can be viewed at wileyonlinelibrary.com]

The client portal was delivered using a high quality user interface within the SilverCloud platform. It provides a homepage, access to the programme content, tools, and other resources. The programme homepage is illustrated in Figure 1.

**FIGURE 2:**
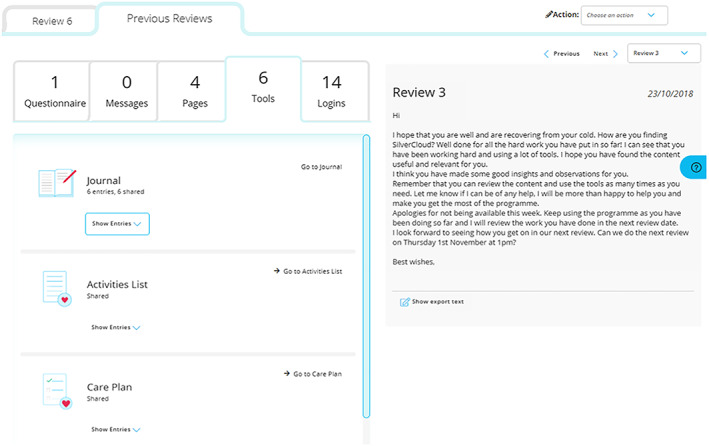
Screenshot of the Clinician dashboard, showing tool usage and review text [Colour figure can be viewed at wileyonlinelibrary.com]

The clinician portal, as illustrated in Figure 2, provides a comprehensive graphical interface to review client engagement and the self‐tracking and early warning information from the service user. Both users and clinicians were able to set reminders for reviewing activity on the platform and a calendar function to set dates and times for reviews.

#### 2.5.3 The Bipolar Toolkit has four core modules

The programme is intended to be self‐paced and service‐users to interact with the various tools as they move through the programme. The ultimate objective of the toolkit is to promote a sense of personal recovery and quality of life in patients with BD who are being treated at secondary care services. The concept of personal recovery is introduced in module 1, and users are asked to set meaningful goals relevant to their own sense of personal recovery. Some of the tools, like the “Care Plan” and the “Medication Log” were specific requests made by on‐site clinicians who thought these tools would be helpful to the patients. A description of the module content and tools is detailed in Table 1.

**TABLE 1:** Description of the programme modules

Module	Content	Tool
1. The facts about bipolar	General information about bipolar, emotions involved and meaning of personal recovery	● Goal Setting tool: Users can set their own goals about personal recovery
		● Care plan: Users can identify problematic areas and determine specific actions to handle them.
		● Journal: Users can introduce daily situations to clarify thoughts and feelings
		● Resources: allows the patient to add helpful web resources
2. Bipolar and me	Available treatments for bipolar, the role of medication, early warning signs, symptoms of bipolar, importance of daily routine	● Medication log: Tool designed to keep track of the medication taken so far and how useful it was.
		● Mood and lifestyle tracker: allows users to track their mood and add associated lifestyle choices.
		● Symptoms of bipolar list: Users can create their own list with the symptoms that characterise their depressive and manic episodes.
		● Early Warning Signs (EWS) Journal: Patients are encouraged to identify their EWS, triggers and how to cope them.
		● Activity Scheduling Tool: a calendar where patients can schedule pleasurable activities.
3. Relationships	The importance of a social network, dealing with fallouts of an episode, relaxation exercises	● Support Network tool: allows users to include different individuals and list them in order of closeness.
		● Crisis plan: encourages users to list the strategies, resources and people that might be available and helpful in a time of crisis.
		● Relaxation exercises: Several audio exercises are included to practice relaxation skills.
4. Sleep	To explain the relationship between sleep and bipolar, sleep hygiene	● Sleep tracker: It is included as a lifestyle choice inside the mood monitor.
		● Tips for sleeping well: Users can create their list of tips to improve their sleep.

### 2.6 Assessments and measures

#### 2.6.1 Feasibility outcomes

Feasibility was assessed from both, patient and clinicians perspectives. For patients, number of recruited patients and engagement with the intervention (i.e., number of logins, time spent, number of tools used, and percentage of programme completion) were gathered. For clinicians, it was gathered the number of clinicians who attended the training, how many of those acted as active suppoorters in the platform, what type of specialists (i.e., nurses, psychiatrists, and occupational therapists), the number of patients supported, and the number of reviews provided.

Satisfaction with treatment (SAT; Richards & Timulak, 2013) is a self‐report measure of participants' positive and negative experiences with the internet‐delivered intervention. Participant responses to statements are rated using a 5‐point Likert scale (1 = disagree very strongly/not at all helpful, 5 = agree very strongly/very helpful).

#### 2.6.2 Semi‐structured interviews for clinicians

It was a semi‐structured interview elaborated ad hoc to explore the experiences of clinicians with the intervention. The interview was composed of two main domains of investigation: usability and acceptability. Questions relating to the usability of the online platform focussed on learnability, efficiency, memorability, error recovery, and clinician satisfaction. Questions relating to acceptability referred to the clinician's opinion on the programme content and the role of the internet‐delivered self‐management tool as an adjunct to treatment as usual for patients with BD.

#### 2.6.3 Semi‐structured interview for patients

It was a semi‐structured interview elaborated ad hoc to explore the experiences of patients with the intervention. The interview was composed of four domains of investigation: changes since starting the online programme, user experience, helpful aspects, and unhelpful aspects.

#### 2.6.4 Psychological validated measures

The Bipolar Recovery Questionnaire (BRQ; Jones, Mulligan, Higginson, Dunn, & Morrison, 2013) was used to assess personal experiences of recovery in BD. Quality of Life in Bipolar Scale (QOL.BD; Michalak & Murray, 2010) assess 12 areas of quality of life in BD. The Brief Illness Perception Questionnaire (BIPQ; Broadbent, Petrie, Main, & Weinman, 2006) measures beliefs about mood swings, and it was specifically adapted for BD (Lobban, Solis‐Trapala, Tyler, Chandler, & Morriss, 2013). The Internal State Scale (ISS, Bauer et al., 1991) assesses mood state severity in four subscales: depression, well‐being, activation, and perceived conflict.

#### 2.6.5 Early warning signs monitoring

This scale was developed ad hoc for the current study. It was composed of two questions where individuals were asked about the frequency of symptoms monitoring of depression and mania. Items were rated on a 4‐point Likert scale from “Never” to “Very regularly.”

### 2.7 Training for clinicians

A 3‐h workshop was conducted with the clinicians that were willing to participate in the study. The workshop was composed of face‐to‐face training using PowerPoint presentations and video tutorials to guide clinicians in the use of the programme. These training materials provided guidance on the solution, how to access and use the intervention, what the clinicians would be able to view of their users' activity, and how to write a review. As the intervention was tool‐driven in nature, supporters were also instructed to consistently encourage their patients to interact with and use the tools. Supporters were encouraged to provide six reviews to their clients over the course of 10 weeks. Each review was expected to take between 10 to 15 min per client. Each site was also assigned an implementation champion, who would oversee and manage the implementation of the programme at the respective sites. At each site, the implementation champion was then responsible for inviting participants to the programme once identified and assigning them to the relevant supporter on the care team. The implementation champion was selected by consensus in each site (a psychiatrist in site A and a community nurse in site B).

### 2.8 Treatment as usual

Stabilized patients with BD are usually treated in community services where they receive outpatient clinical reviews, continuation of therapies that have commenced or are referred to other services. Some of these therapies include sessions with an occupational therapist or social worker if the episode has impacted work and social/family life. Bipolar support groups offer additional patient support. Patients typically attend outpatient clinics for monitoring of mental state and any medication compliance/side effects.

### 2.9 Procedure

Patients who met the inclusion criteria were offered the opportunity to participate in the trial through a member of their care team, who described to patients what the study would entail for them, the aims, rationale and provided them with the participant information sheet. Patients were encouraged to read the information sheet carefully and ask any questions they might have. Once patients created a user account, they were then required to provide their informed consent via digital signature to progress and use the intervention. Thereafter, they were sent a welcome message by their supporter and informed that they would be offered six online reviews over a period of 10 weeks. Patients also completed psychometric assessments at baseline and post‐intervention timepoints. Once the 10‐week supported period ended, participants retained access to the intervention in a self‐guided modality for an indefinite period of time (lifelong access). Patient participants in the trial were invited to participate in a semi‐structured interview regarding their experience of the use of the online self‐management tool. During the intervention period, the research team arranged weekly calls with the clinicians at each site to supervise that the intervention was deployed as expected (i.e., reviews were completed timely, and the content was appropriate) and the progress of recruitment. Clinicians who supported participants in the trial were also contacted to participate in a semi‐structured interview regarding their views on the feasibility and acceptability of the toolkit. All adverse events were to be handled by the in‐house care team according to normal service procedures.

### 2.10 Data analysis

The feasibility data regarding recruitment objectives were analysed by comparing the target objectives (i.e., 10 patients per site) to those achieved in the study and by comparing the number of clinicians trained in the use of the intervention to those who actively supported participants. The intended use of the programme was analysed by comparing the review timeline with the average number of reviews delivered by supporters and associated delays in their delivery. To examine feasibility and acceptability further, usage information will also be illustrated to determine the average use of the platform in terms of number of logins, time spent on the platform, tools used, and percentage completion of the programme. To examine acceptability, data from the SAT questionnaire were descriptively analysed by calculating the mean score and standard deviation per item, which was then tabulated. Further examining acceptability through the qualitative data from the clinician and participant semi‐structured interviews was analysed using the descriptive–interpretative method (Elliott & Timulak, 2005). Preliminary analysis showed that the outcomes data were not normally distributed across the sample; therefore, Wilcoxon signed‐rank tests were conducted on the primary and secondary psychological outcomes to analyse median changes in the scores across pre‐post intervention timepoints. In the same vein, differences in usage between completers and noncompleters were explored through *U*‐Mann Whitney non‐parametric analyses. All analyses were conducted in SPSS 24.

## 3 RESULTS

### 3.1 Feasibility outcomes

#### 3.1.1 Recruitment

**FIGURE 3:**
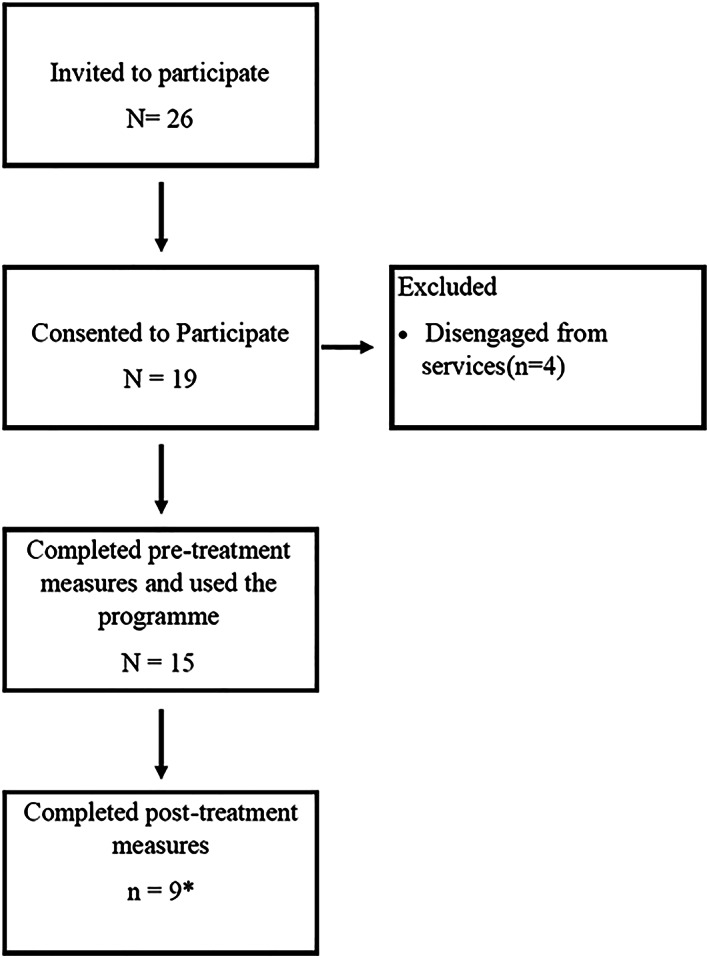
Flowchart of the study. ^*^ Individuals did not provide data on the Brief Illness Perception Questionnaire (BIPQ) and the satisfaction questionnaire, and one individual did not provide data on the Bipolar Recovery Questionnaire (BRQ) and BQOL

Patient recruitment for the study was originally planned to take place over a 1‐month period (September 2018), but due to delays in recruitment at the sites, it was extended to 2 months (September–October 2018). Across two sites, 26 patients were invited to participate in the trial. Ninteen (*N* = 19) patients accepted the trial invitation and consented to participate. Fifteen (*N* = 15) participants (seven from site A and eight from site B) completed the required pretreatment research measures. Of those who did not complete the post‐treatment questionnaires, one individual was hospitalized due to relapse in bipolar symptomatology. A flowchart summarizing this information is illustrated in Figure 3. Table 2 illustrates the sociodemographic characteristics of the sample.

**TABLE 2:** Sociodemographic characteristics of the sample

Variable	Frequency	Percentage (%)
Age (*M,* SD)	40.23 (12.02)	
Gender (*n* = 13)		
Female	3	76.9
Male	10	23.1
Education (*n* = 13)		
High school	4	30.8
Undergraduate	4	30.8
Other certificate	4	30.8
None	1	7.7
IT literacy (*n* = 11)		
Very confident	4	36.4
Confident	2	18.2
Average	3	27.3
Mildly confident	2	18.2
Employment (*n* = 12)		
Student	1	8.3
Part‐time	1	8.3
Full‐time	2	16.7
Unemployed	1	8.3
Retired	1	8.3
At home parent	6	50
Marital status (*n* = 12)		
Have partner	3	25
Married	3	25
Separated	1	8.3
Single	5	41.7
Diagnosis (*n* = 11)		
Bipolar I disorder	4	36.4
Bipolar II disorder	4	36.4
Other^a^	3	27.3

^a^ All participants were referred by their clinical team as being diagnosed with Bipolar disorder. Still, three of them did not report a specific bipolar disorder diagnosis in the sociodemographic questionnaire.

Clinicians were trained in the use of the intervention approximately 1 month prior to the start date of patient recruitment. In regard to clinician uptake, 11 clinicians were trained at site A and a further 11 at site B for a total of 22 supporters. Of these 22, nine (four at site A and five at site B) became “active supporters” with assigned clients on the platform. The spread of patients assigned to supporters at Site A was skewed, with one clinician being responsible for five patients, two responsible for one patient, and one clinician never being assigned a client. Site B had a more even distribution, with three clinicians being responsible for one patient, one for two patients, and the last clinician being responsible for three patients. All clinicians who became active supporters were either consultant psychiatrists or community nurses.

### 3.2 Provision of reviews

Clinicians at both sites were instructed to provide participants with six reviews over a 10‐week period. Overall, supporters offered an average of 3 (standard deviation [SD] = 1.6) reviews per participant. Splitting the number of reviews per site, Site A (*M* reviews_(min‐max)_ = 2_(0–3)_, SD = 1.15) provided fewer reviews per participant than site B (*M* reviews_(min‐max)_ = 3.88_(2–6)_, SD = 1.15). Only one participant across the total sample (from site B) received the intended number of reviews over the 8‐week supported period.

#### 3.2.1 Platform usage

Usage data for the entire sample are presented in Table 3. Medians are also included in the table to more clearly illustrate the platform usage of the sample, as the high usage of certain participants would have inflated mean scores in this instance. Mann–Whitney *U* tests were conducted to explore the presence of significant differences between completers and noncompleters in terms of usage. Results showed significant differences in terms of log‐ins (Mann–Whitney *U* = 3.50, *p* = .003), session length (Mann–Whitney *U* = 8.00, *p* = .026), tool usage (Mann–Whitney *U* = 6.50, *p* = .012), and module 2 completion (Mann–Whitney *U* = 8.00, *p* = .023). Mann–Whitney *U* tests were also conducted to test differences in terms of usage between sites, and only number of reviews showed significant differences between groups (Median Site A _(min‐max)_ = 4_(2–6)_, Median Site B _(min‐max)_ = 2_(0–3)_, Mann–Whitney *U* = 9.5, *p* = .029).

**TABLE 3:** Platform usage by participants completion and total sample

Variable	Completers (*n* = 9)			Noncompleters (*n* = 6)			Total (*N* = 15)		
	*M* (min‐max)	*SD*	Median	*M* (min‐max)	*SD*	Median	*M* (min‐max)	*SD*	Median
Number of logins	23.44 (5–86)	24.85	14	5.33 (2–9)	2.58	5.50	16.2 (2–86)	20.98	11.00
Number of reviews	3.67 (1–6)	1.58	4.00	2.00 (0–3)	1.09	2.00	3 (0–6)	1.60	3.00
Number of tools used^a^	65.11 (3–212)	60.94	42.00	13.33 (0–38)	13.79	12.15	44.4 (0–212)	53.66	35.00
Time spent (h)	3.49 (1.47–6.95)	1.89	3.12	1.38 (.07–2.78)	1.09	1.25	2.65 (.07–6.95)	1.90	35.00
Module 1 completion	58.02% (0%–100%)	45.06%	66.67%	25.93% (0%–100%)	42.55%	0.00%	45.18% (0%–100%)	45.52%	44.44%
Module 2 completion	74.75% (18.18%–100%)	32.07%	90.91%	31.82% (0%–90.91%)	31.88%	27.27%	57.58% (0%–100%)	45.45%	37.74%
Module 3 completion	44.44% (0%–100%)	44.29%	28.57%	21.43 (0%–71.43%)	33.50%	0.00%	35.24% (0%–100%)	40.72%	14.29%
Module 4 completion	55.56% (0%–100%)	52.70%	100%	19.44% (0%–100%)	40.02%	0.00%	41.11% (0%–100%)	49.95%	0.00%
Total programme completion	60.27% (6.06%–100%)	36.09%	66.67%	25.76 (0%–72.73%)	27.82%	13.63	46.46% (0%–100%)	36.43%	42.42%

^a^ Tools can be used repeatedly.

### 3.3 Acceptability outcomes

#### 3.3.1 Patient satisfaction

Seven participants completed the SAT questionnaire. Five (*n* = 5, 71.4%) of the patients rated the programme as being a little better or much better compared with other similar treatments. Four (*n* = 4, 57.1%) reported being happy using the computer to access the programme and found the programme easy to use. Three of them (*n* = 3, 42.9%) strongly agreed that they would continue using the self‐management programme, whereas the other three (*n* = 3, 42.9%) were neutral about continuing using it. Four of the patients (*n* = 4, 71.4%) agreed strongly or very strongly that they would recommend the programme to other patients dealing with the same condition. Lastly, five of the seven users (*n* = 5, 71.4%) found the programme quite or very helpful.

### 3.4 Qualitative interviews

Three patients participated in qualitative interviews, and demographic information is presented in Table 4.

Patients reported changes due to their engagement with the programme, including increased awareness of symptoms and self‐management approaches, for example, the programme was

**TABLE 4:** Demographic information of patients participating in qualitative interviews (*N* = 3)

Patient	Gender	Age	Computer literacy
P1	Male	66	Can do what he needs to
P2	Female	46	Regularly uses the Internet/technology
P3	Female	40	Not overly tech savvy

clear on symptoms and stuff like that … with things I would have forgotten … and I found the charts good to put in the mood and (realise) I'm kind of feeling this way, or this way ( P3).

Two participants reported feeling empowered and confident, for example,

it's been very good. Very positive. And it shows me where I'm at as well (in terms of my recovery; P2).

Participants' reports on helpful content were described as reinforcing existing knowledge, and mentioned that personal stories felt relatable to the participants' experiences of BD. For example,

… I really like reading other people's experiences and stuff like that and I can relate to things ( P3).

They also commented on the importance of the support they received from their caregivers

… My own doctor is involved in it … He reassured me … how reliable it is as a service. So, I went with it [the online self‐management programme] ( P2).

All three participants reported that there were areas of self‐management which the programme had no effect on, specifically describing no effect on sleeping habits or mood swings. Some negative aspects were also reported, specifically relating to the memorability of how to use the programme and difficulties navigating the platform, for example,

… It was once I went off it, it seemed to get hard to get back in to where I left off ( P1).

All participants agreed that there wasn't enough contact with the supporter.

… I would not have been happy with that [supporter contacts] at all … I think that contact wants to be personal … ( P1).

One participant repeatedly reported that the programme added nothing to their understanding and self‐management of BD,

… like it was boring … I've known everything that was on it really ( P1).

In general, participants' attitudes towards online interventions were mixed, some positive,

… I have any extra back up of a service as really benefitting my bipolar you know ( P2).

Others less than positive, for example,

… I would be in total disagreement with the whole system like, of online … ( P1).

Three clinicians participated in qualitative interviews, and their demographic information is presented in Table 5. All clinicians reported that the programme was straight‐forward to navigate and enjoyable to use, for example,

**TABLE 5:** Demographic information of clinicians participating in qualitative interviews (*N* = 3)

Clinician	Gender	Role on clinical team	Computer literacy
C1	Male	Psychiatrist	Confident
C2	Female	Nurse	Confident
C3	Female	Nurse	Confident

I did not find any … difficulties with it ( C3).

In particular, the online intervention satisfied their criteria for a bipolar self‐management programme and met clinician and care needs, for example,

I like the package itself … I am … very positive about it ( C1).

Two of three clinicians reported that the online programme was an acceptable adjunct to treatment as usual, for example,

… I think for the people again who, like are coming every three months or so [to clinics], I think it's a useful way of maybe keeping a little bit of an eye on things ( C1); … we do our own separate groups for management and for mindfulness … it does interlink with what we are doing on this ( C2).

All participants commented positively on the utility of the content and self‐management tools in the programme, including tools such as goal setting, mood monitoring, and mindfulness exercises, for example,

… we would always recommend the relaxation, the mindfulness piece, the body scans … it was nice to have a page where everything was on it ( C2).

All clinicians reported on the positive aspects of supporting their clients through reviews, for example,

… with the reviews you are not necessarily responding to someone coming in and saying, or writing down, I feel like this … You're responding to specific things they have done ( C1).

Two clinicians reported difficulties in prioritizing patient reviews due to crisis and subsequently missing or being late with their reviews, for example,

… Like emergency situations would come up that would make that (reviews) difficult a few times to fulfil on time ( C1).

Reported barriers to treatment success included access to technology and electronic health records, which effected the smooth implementation of the programme, for example,

… if it was something that was … more integrated in to a system which does not exist obviously in the HSE, then I think it would have been a more streamlined process … and may have also been more able to link in with the clinical issues when they came up ( C1).

Two clinicians commented on the inclusion criteria for the trial and intervention, for example,

… I think … if I was doing it again, I'd probably be stricter on the criteria of maybe there's a sort of definite period since last admission … I think something like that would have probably reduced the numbers of people (dropping‐out, C1); … I had somebody who was 30 years diagnosed who found it very repetitious. It's the information she'd heard numerous times so did not particularly like that ( C2).

### 3.5 Outcomes data

Results for the different clinical measures can be observed in the Table 6. Wilcoxon analyses showed significant differences in median scores from pre‐ to post‐intervention were observed for the BRQ (*z* = 2.38, *p* = .017). Wilcoxon analyses were also carried out for the individual items on the BIPQ (they cannot be combined), and a significant difference in median scores was observed on item 4 (“How much do you think your treatment can help with your mood swings?”; scale range from 0 to 10) from pre‐ (7) to post‐intervention (8), *z* = 1.98, *p* = .047. No differences were found for the other items on the BIPQ. Analyses on other quantitative outcomes (QOL.BD, Early Warning Signs [EWS], and ISS) did not achieve statistical significance.

**TABLE 6:** Pre and post median scores for main outcomes

	Median			Change (*n*)			Wilcoxon statistics	
	Pre	Post	Difference	Positive	Negative	No difference	*z*	*p*
BRQ (*n* = 8)	2,249	2,525	249.5	7	1	0	2.38	.017
QOL.BD (*n* = 8)	40	39.5	3.5	6	2	0	1.76	.79
EWS (depression) (*n* = 9)	3	2	0	2	3	4	−0.707	.48
EWS (mania; *n* = 9)	2	2	0	2	1	6	0	1
ISS (activation; *n* = 9)	92	56	−10	4	5	0	−0.415	.678
ISS (depression index) (*n* = 9)	18	17	−1	2	5	2	−1.352	.176
ISS (perceived conflict) (*n* = 9)	106	46	−17	3	6	0	−0.889	.374

Abbreviations: BRQ, Bipolar Recovery Questionnaire; EWS, Early Warning Sign; ISS, Internal State Scale; QOL.BD, Quality of Life in Bipolar Scale.

## 4 DISCUSSION

The current project sought to explore the feasibility, acceptability, and preliminary efficacy of integrating an internet‐delivered self‐management programme within the treatment pathway for patients with BD who were attending services at two secondary‐care services in Ireland. This study builds upon previous literature on digital interventions for bipolar by exploring the utility of these interventions when integrated into existing outpatient services and the experiences from patients and the staff involved in the care of these patients Overall, the intervention was found acceptable, and patients and healthcare staff found the intervention easy to use and convenient. The intervention produced no significant deterioration in regard to the assessed outcomes, and improvements in participants' sense of personal recovery were found. In regard to feasibility, several obstacles and barriers were identified in the implementation of the intervention within the care pathways, which we discuss.

In terms of recruitment and uptake from patients, 57% of participants accepted the invite and completed the baseline measures, and 34% completed the post‐treatment measures. The observed completion rates are lower than the ones observed in previous trials of internet‐delivered interventions for bipolar (Lobban et al., 2017; Todd et al., 2014). These differences could be explained by the fact that previous studies were conducted in community settings were individuals self‐referred to the services and, therefore, might be more motivated, whereas in this study patients were referred by clinicians from specialized services. Thus, taking into account that this trial was conducted in a service setting with no previous experience of digital interventions, and largely inexperienced in research, these recruitment rates and research retention rates can be understood relatively positively. A longer recruitment period may have resulted in higher numbers of participants in the trial. In this sense, prior studies had recruitment periods between 12 to 34 months, although they were aiming for larger sample sizes (*n* > 100) and they were conducted in community settings, which makes results hardly comparable (Lobban et al., 2017; Proudfoot et al., 2012; Todd et al., 2014).

In regard to the uptake from clinicians, less than half of those trained became active supporters on the platform, and the average number of reviews provided was below the recommendations. Clinicians reported not being able to provide the recommended amount of reviews due to their significant workload and also due to the complexities of the services in which they work. As mentioned by clinicians in the interviews, and also observed by the researchers, the solution was not properly integrated into the service and was, therefore, not considered a priority task. Instead, it was taken as an add‐on to the their current workload, and when other priorities or emergency situations popped up, reviews were skipped which, ultimately, impacted on patient engagement. Therefore, even though participants involved in this study and previous reviews (Aref‐adib et al., 2018; Hidalgo‐mazzei et al., 2015) indicate the value of healthcare staff acting as facilitators on these interventions, processes need to be built around the service pathways to ensure that reviews are considered an essential task within the workload, similar to attending regular visits. These results are in line with the cumulative evidence on implementation research, which indicates that for a successful implementation, digital interventions need to be interoperable within existing systems and integrated fully into service pathways (Aref‐adib et al., 2018). Despite the implementation issues that prevented clinicians from offering more reviews, they found the intervention easy to use and navigate, which is key for a proper deployment of the solution given the busy caseloads they have to work on. They also found the content and self‐management tools useful and convenient as an adjunct to treatment as usual, because it complemented some of the work they were doing in parallel.

The usage of the intervention was adequate, and there was a high frequency of tool usage, compared with previous studies on this platform (Enrique, Palacios, Ryan, & Richards, 2019), which emphasizes the tool‐driven nature of this programme (Naslund, Marsch, McHugo, & Bartels, 2015). Usage levels were significantly higher for those who completed the post assessment, which is in line with prior studies (Van Den Heuvel et al., 2018). The mood monitor was highlighted as one of the most valued tools, because it helped patient to gain awareness about symptoms and other studies have also outlined the relevance of similar tools (Hidalgo‐mazzei et al., 2018; Van Den Heuvel et al., 2018). Overall, the usage levels were particularly good considering the minimal number of reviews patients received. In this sense, receiving the recommended amount of reviews might have enhanced the uptake.

In regard to patient satisfaction, the relatively small number of completer participants found the programme easy to use, liked the idea of the programme being online, found it helpful, and would recommend it to others, which is in line with prior findings on acceptability (Naslund et al., 2015; Van Der Krieke, Wunderink, Emerencia, De Jonge, & Sytema, 2014). Despite the general acceptability, patients and clinicians agreed that the content did not always fit with the users needs, especially for those with many years dealing with the condition. This raises the question of for whom digital interventions are most suitable for. BD is considered a very complex condition and individual preferences need to be accounted where some patients might prefer nondigital alternatives (i.e., paper and pencil printable materials) and these should be made available for these individuals (Proudfoot et al., 2012). In this sense, the ultimate aim of integrating digital within specialized services must be to enhance clinician's reach by providing accessible and timely interventions and not to replace conventional services. Future studies should explore whether implementing digital solutions helps to reduce the service burden and the use of resources through cost‐effectiveness and utility analyses (Lobban et al., 2017).

Regarding the effects of the intervention on the measures included, the results showed significant changes from pre to post for personal recovery. Given the fact that the programme was developed under the umbrella of the personal recovery approach, the results suggest that the programme served its purpose in this regard. The benefits observed in terms of personal recovery align with the reports of the patients, who stated they improved in their awareness and understanding of the condition, and also felt more empowered. However, these results must be taken with caution given that this study was not powered to detect clinical differences. No significant gains were observed for quality of life, beliefs about mood swings, or severity of symptoms. These results are not surprising, because several studies conducted in this field have shown mixed results (Gliddon et al., 2019; Van Den Heuvel et al., 2018), indicating the need for controlled and well‐powered studies (Naslund et al., 2015). Furthermore, the current intervention was brief in its usage, and in the context of the management of a chronic long‐term condition, it is probably not surprising that no significant changes were reported and future studies should deploy these interventions for longer periods.

The main strength of this study is that the intervention was integrated in specialized treatment services, which allowed for observations to be made in regard to the implementation factors that should be considered for a successful integration into existing services. Furthermore, the mixed methods evaluation allowed the authors to better understand the results obtained from the quantitative data. The study also presents several limitations. First, no control group was included in the trial, which prevented us to attribute the changes observed to the intervention, although the absence of changes in the other measures may indicate otherwise. Second, given the specificities of the sites and the short period of recruitment, a small sample size was included in the trial. Future studies should include a control condition and well‐powered samples that allow for confirmatory analysis about the effects of these interventions. Implementation issues that arose during the trial (i.e., insufficient support) could have undermined the effects of the intervention by reducing the uptake and, therefore, effecting the observed benefits. Third, the current study only included eutimic outpatients and did not explore how the intervention could help those experiencing acute symptoms of bipolar. This caveat limits the implementation learnings of the study, because it is not possible to determine if the solution may in pathways for these patients. Future studies should look at this cohort to explore if similar findings are observed. Fourth, the sample included in this study was composed mostly of male patients, which may have skewed the results but this would be unlikely, because evidence suggest that gender has limited effect on treatment response (Arnold, 2003). Fifth, the ability of the study to report on incidences of adverse events was limited due to ethical constraints; the research team was not able to access the care records of those involved with the study and were also unable to receive this information from the care team and service. Future research should account more completely report on adverse events and trends associated with these in the dataset. Lastly, acceptability and clinical impact was only assessed for those who completed post‐treatment outcomes, which could be biasing the results, so that those who remained in the trial had a more positive attitude towards the intervention. Future studies with larger samples should account for missing data in the analyses in order to reduce the influence of this bias in the outcomes.

## 5 CONCLUSION

This is the first study that tests the feasibility of a digital self‐management intervention for patients with BD in public mental health services in Ireland. The findings showed that the intervention was accepted well by patients and clinicians, with the uptake of the intervention being high. However, contextual factors need to be accounted for in order to effectively deploy the intervention in future iterations. Thus, more implementation research is needed in order to increase the understanding of how to promote the integration and the uptake of digital interventions for individuals with severe mental health conditions within routine care settings.

## DISCLOSURE OF INTEREST

A. E., D. D., K. L., and D. R. are employees of SilverCloud Health, a private limited company that works in the development and implementation of digital interventions for mental health. A. E., D. D., and D. R. are also researchers with the E‐Mental Health Research Group of Trinity College Dublin (TCD) and adhere to both the research ethics frameworks and policy on good research practice of TCD. S. J. is a professor of Clinical Psychology in the Faculty of Health and Medicine at Lancaster University, United Kingdom which received funding from SilverCloud Health for his input as a subject matter consultant to provide the relevant expertise and research guidance for the Bipolar Lighthouse Project.

## AUTHOR CONTRIBUTIONS

A. E. and D. R. conceptualized the initial design of the trial with input from S. J. A. E. led on the development of the first and subsequent drafts of the manuscript, with significant contributions from D. D., K. L., D. R., and S. J. K. L. actioned the qualitative segment of the study. A. E., D. D., and K. L. trained clinicians supporting in the study in regard to both research procedures and intervention administration. A. E. and D. D. provided continuous research support to all sites involved with the project.
